# Implementation and Accuracy of BinaxNOW Rapid Antigen COVID-19 Test in Asymptomatic and Symptomatic Populations in a High-Volume Self-Referred Testing Site

**DOI:** 10.1128/Spectrum.01008-21

**Published:** 2021-12-01

**Authors:** Zishan K. Siddiqui, Mihir Chaudhary, Matthew L. Robinson, Anna B. McCall, Ria Peralta, Rogette Esteve, Charles W. Callahan, Yukari C. Manabe, James D. Campbell, J. Kristie Johnson, Maryam Elhabashy, Melinda Kantsiper, CONQUER COVID Consortium, James R. Ficke

**Affiliations:** a Department of Medicine, The Johns Hopkins University School of Medicine, Baltimore, Maryland, USA; b Department of Surgery, University of California-East Bay, Oakland, California, USA; c Baltimore Convention Center Field Hospital, Baltimore, Maryland, USA; d Population Health, University of Maryland Medical System, Baltimore, Maryland, USA; e Center for Vaccine Development and Global Health, Department of Pediatrics, University of Maryland School of Medicine, Baltimore, Maryland, USA; f Division of Hospital Medicine, The Johns Hopkins Bayview Medical Center, Baltimore, Maryland, USA; g Department of Orthopedic Surgery, The Johns Hopkins University, Baltimore, Maryland, USA; h University of Maryland Baltimore County, Catonsville, Maryland, USA; i Department of Pathology, University of Maryland School of Medicine, Baltimore, Maryland, USA; Johns Hopkins Hospital

**Keywords:** COVID-19, field hospital, point-of-care testing, rapid antigen testing, reverse transcription PCR, SARS-CoV-2, sensitivity and specificity

## Abstract

Rapid antigen tests are simple to perform and provide results within 15 min. We describe our implementation and assess performance of the BinaxNOW COVID-19 Antigen Test (Abbott Laboratories) in 6,099 adults at a self-referred walk-up testing site. Participants were grouped by self-reported COVID-19 exposure and symptom status. Most (89%) were asymptomatic, of whom 17% reported potential exposure. Overall test sensitivity compared with reference laboratory reverse-transcription [RT] PCR testing was 81% (95% confidence interval [CI] 75%, 86%). It was higher in symptomatic (87%; 95% CI 80%, 91%) than asymptomatic (71%; 95% CI 61%, 80%) individuals. Sensitivity was 82% (95% CI 66%, 91%) for asymptomatic individuals with potential exposure and 64% (95% CI 51%, 76%) for those with no exposure. Specificity was greater than 99% for all groups. BinaxNOW has high accuracy among symptomatic individuals and is below the FDA threshold for emergency use authorization in asymptomatic individuals. Nonetheless, rapid antigen testing quickly identifies positive among those with symptoms and/or close contact exposure and could expedite isolation and treatment.

**IMPORTANCE** The BinaxNOW rapid antigen COVID-19 test had a sensitivity of 87% in symptomatic and 71% asymptomatic individuals when performed by health care workers in a high-throughput setting. The performance may expedite isolation decisions or referrals for time-sensitive monoclonal antibody treatment in communities where timely COVID PCR tests are unavailable.

## INTRODUCTION

The coronavirus disease 2019 (COVID-19) pandemic, caused by severe acute respiratory syndrome coronavirus 2 (SARS-CoV-2), continues to threaten global public health. Easily accessible and widely distributed SARS-CoV-2 testing is a cornerstone of limiting the spread of the virus globally even as vaccines are distributed. The U.S. federal government continues to invest in SARS-CoV-2 testing, which remains essential as we begin to emerge from the pandemic (1).

Reverse transcription–PCR (RT-PCR) remains the “gold standard” for identifying infected individuals (2). However, such testing can take more than 48 h to yield results (3). According to a large, nationally representative survey sample, median test result wait times are longer for African Americans (5 days) and Hispanic Americans (4.6 days) compared with white Americans (3.9 days) (3). Rapid lateral flow antigen (herein, “rapid antigen”) tests detect viral proteins and many have a 15-min turnaround time for results, which can enable individuals who test positive to quickly self-isolate to prevent transmission and can accelerate assessment of individuals at high risk for severe COVID-19 for possible monoclonal antibody treatment. Fourteen brands of such tests have received emergency use authorization by the U.S. Food and Drug Administration for clinical use in symptomatic individuals, and three have been approved for home use (4, 5). During February 2021, the U.S. government invested more than $800 million to increase the production of point-of-care tests, such as rapid antigen tests (1, 6).

Although studies have evaluated the performance of rapid antigen testing, to our knowledge only a few have been evaluated in high-throughput settings, and results from these studies have been inconsistent (7–9). Manufacturer-reported data are based on symptomatic patients and small sample sizes and, until recently, lacked transparency regarding exact RT-PCR test used as the comparator (10). Therefore, we evaluated sensitivity and specificity, as well as our implementation of a rapid antigen test (BinaxNOW COVID-19 Antigen Test [herein, “BinaxNOW rapid antigen test”], Abbott Laboratories, Abbott Park, IL) at a high-volume COVID-19 testing site and compared its performance in symptomatic versus asymptomatic patients, as well as by self-reported COVID-19 exposure in asymptomatic individuals.

## RESULTS

A total of 9,866 adults underwent RT-PCR testing between December 23, 2020 and January 11, 2021. Of these, 6,099 (61%) enrolled in this study of which 6,061 had valid rapid antigen testing and RT-PCR results. Among the study sample, 3,223 participants (53%) were female, 3,743 (61%) were white, 1,110 (18%) were African American, and the median age was 31 years (interquartile range [IQR], 26–41 years). We obtained symptom status data from 6,098 participants, 5,452 (89%) of whom were asymptomatic. Symptomatic participants were slightly younger (34 vs. 35 years) (*P*  =  0.04) and more frequently non-white Hispanic (5.9% vs. 3.7%) (*P*  <  0.01) (Table 1).

**TABLE 1:** Demographic characteristics for 6,099 adult participants at a self-referred walk-up COVID-19 mass testing site

Characteristic	*N* (%)	
	Asymptomatic (*n* = 5,451)^a^	Symptomatic (*n* = 646)^a^
Female sex	2,858 (52)	365 (57)
Race/ethnicity		
African American	987 (18)	123 (19)
Asian	417 (7.6)	39 (6.0)
Non-white Hispanic	199 (3.7)	38 (5.9)
White	3,363 (62)	380 (59)
Other	485 (8.9)	66 (10)
Age, years	31 (26–41)^b^	31 (26–40)^b^

a Demographic data are missing for two patients.

b Data presented as median (interquartile range).

Among asymptomatic participants, 145 (2.7%) reported living with someone with confirmed or suspected COVID, 498 (9.1%) reported being within 6 feet of someone with confirmed or suspected COVID-19 for >15 min, and 248 (4.5%) reported other exposure types. The COVID-19 prevalence rate was 3.7% (222/6,061) overall, 1.6% (87/5,418) for asymptomatic individuals, and 21% (135/642) for symptomatic individuals. Among those reporting any exposure, the median number of days since exposure was 5 (IQR, 3–7). For symptomatic participants, the median number of days since symptom onset was 3 (IQR, 4–5), and 89% (573/642) of participants were tested ≤7 days after symptom onset.

### Quality control.

**FIG 1:**
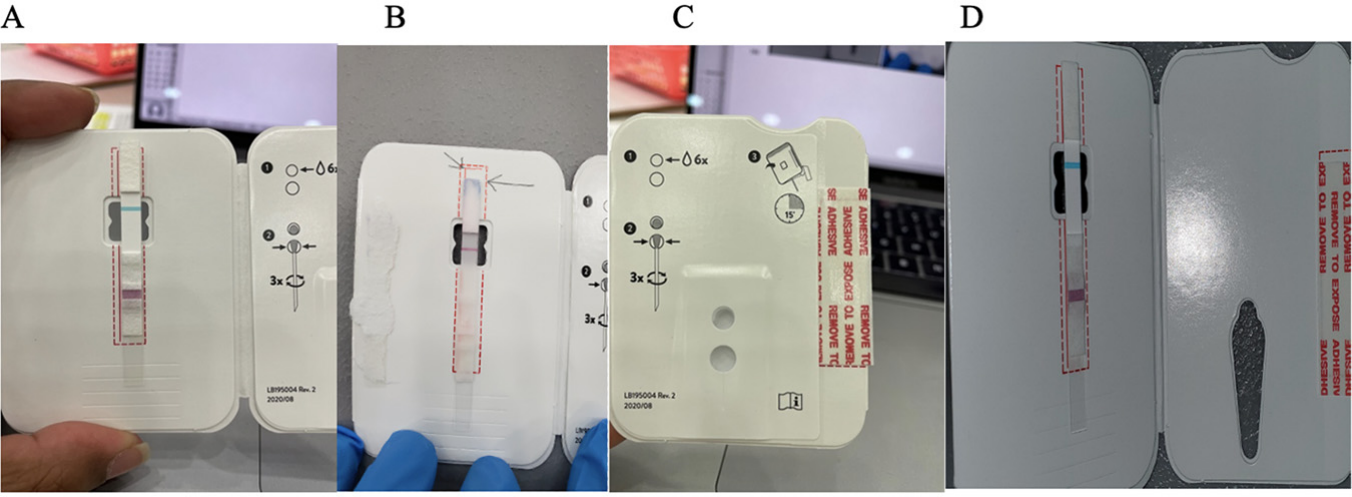
Photographs showing BinaxNOW COVID-19 antigen test kit errors. (A) Normal strip alignment. (B) A misaligned strip. (C) A normal well to insert swab with sample. (D) A missing well.

Rapid antigen test quality control was performed during six testing days during which 3,806 rapid antigen tests were completed. Random quality control checks were performed for 120 tests with negative results, 85 with positive results, and 24 with indeterminate results. Concordance between the primary test reader and the expert tester was 100% for each of the 120 test results reviewed. A total of 33 test results (0.5%) were indeterminate, and 30 test cards were not used because of kit errors. Types of kit errors included missing wells and misaligned test strips (Fig. 1).

### Test accuracy.

Sensitivity of all rapid antigen tests was 81% (95% CI 75%, 86%), specificity was 99.8% (95% CI 100%, 100%), positive predictive value was 93% (95% CI 89%, 96%), and negative predictive value was 99% (95% CI 99%, 100%). The sensitivity of the rapid antigen test was higher in symptomatic participants (87%; 95% CI 80%, 91%) than for asymptomatic participants (71%; 95% CI 61%, 80%) (*P*  <  0.01). The sensitivity of the test was 80% (95% CI 44%, 97.5%) for those who reported living with someone with confirmed or suspected COVID-19; 73% (95% CI 45%, 92%) for those who reported having been within 6 feet for >15 min of someone with confirmed or suspected COVID-19; 100% (95% CI 70%, 100%) for those who reported any other exposure; and 64% (95% CI 51%, 76%) for those who reported being unexposed. Among asymptomatic participants, the positive predictive value was 90% (95% CI 80%, 96%), and among symptomatic participants the positive predictive value was 95% (95% CI 90%, 98%) (Table 2). The specificity was 99% for symptomatic participants and >99% for asymptomatic participants among all exposure groups.

**TABLE 2:** COVID-19 rapid antigen test accuracy rates using RT-PCR as “gold standard” for adult participants at a self-referred walk-up COVID-19 mass testing site^a^

Exposure group	Positive/total (%)		Positive/total (%)	
	RT-PCR	Rapid antigen test	Sensitivity	Specificity
Overall	222/6,061 (3.7)^b^	192/6,061 (3.2)	179/222 (81)	5826/5,839 (100)
Symptomatic	135/642 (21)	123/642 (19)	117/135 (87)	501/507 (99)
Symptoms ≤7 days	127/573 (22)	116/573 (20)	112/127 (88)	442/446 (100)
Symptoms >7 days	8/65 (12)	7/65 (11)	5/8 (63)	55/57 (97)
Asymptomatic	87/5,418 (1.6)	69/5,418 (1.3)	62/87 (71)	5,324/5,331 (100)
With no exposure	53/4,313 (1.2)	34/4,313 (0.8)	34/53 (64)	4,257/4,260 (100)
With any exposure	34/886 (3.8)	28/886 (3.2)	28/34 (82)	848/852 (100)
Living with confirmed or suspected COVID-19–positive person	10/144 (6.9)	8/144 (5.6)	8/10 (80)	133/134 (99)
Within 6 feet of confirmed or suspected COVID-19–positive person for >15 min	15/495 (3.0)	11/495 (2.2)	11/15 (73)	478/480 (100)
With other exposure	9/247 (3.6)	9/247 (3.6)	9/9 (100)	237/238 (100)
Any exposure group within 5 days of exposure^c^	17/469 (3.6)	19/469 (4.1)	16/17 (94)	449/452 (99)
African American	60/1,100 (5.5)	48/1,100 (4.4)	45/60 (75)	1,037/1,040 (100)
Symptomatic	27/121 (22)	23/19 (12)	22/27 (81)	93/94 (99)
Asymptomatic	33/979 (3.4)	25/979 (2.6)	23/33 (70)	944/946 (100)
Non-white Hispanic	23/236 (9.8)	21/236 (8.9)	20/23 (87)	212/213 (100)
Symptomatic	17/38 (45)	14/38 (37)	14/17 (82)	21/21 (100)
Asymptomatic	6/198 (3.0)	7/198 (3.5)	6/6 (100)	191/192 (99)
Female sex	93/3,206 (2.9)	79/3,206 (2.5)	76/93 (82)	3,110/3,113 (100)
Symptomatic	64/361 (18)	56/361 (16)	55/64 (86)	296/297 (100)
Asymptomatic	29/2,845 (1.0)	23/2,845 (0.8)	21/29 (72)	28,14/2,816 (100)
Male sex	129/2,852 (4.5)	113/2,852 (4.0)	103/129 (80)	2,713/2,723 (100)
Symptomatic	71/281 (25)	67/281 (24)	62/71 (87)	205/210 (98)
Asymptomatic	58/2,570 (2.3)	46/2,570 (1.8)	41/58 (71)	2,507/2,512 (100)
CT count <25^d^	117/117(100)	113/117 (97)	113/117 (97)	0/0 (0)
Symptomatic	74/74 (100)	72/74 (97)	72/74 (97)	0/0 (0)
Asymptomatic	43/43 (100)	41/43 (95)	41/43 (95)	0/0 (0)

a CT, cycle threshold; RT-PCR, reverse transcription–PCR.

b Of 6,099 tests, 38 were indeterminate/missing, for a total of 6,061 test results analyzed.

c Median (interquartile range) time since exposure was 5 (3–7) days.

d Median CT count for all participants was 25.

**FIG 2:**
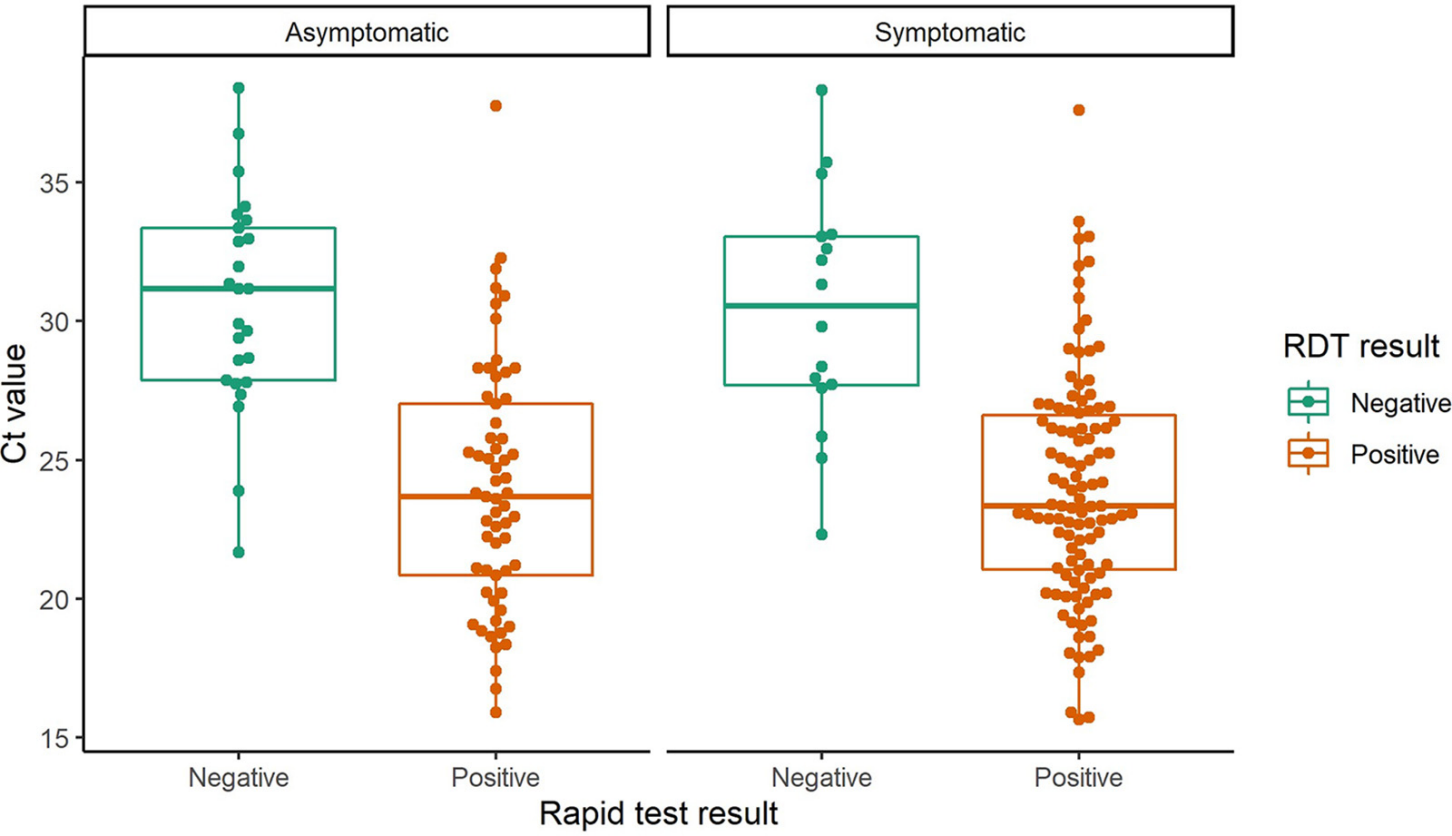
Box plot showing RT-PCR cycle threshold values and COVID-19 rapid antigen test results for symptomatic and asymptomatic participants. Mean cycle threshold (CT) count (negative vs. positive, *P value*): asymptomatic (24.0 vs. 30.9, *P* < 0.0001); symptomatic (24.0 vs. 30.4, *P* < 0.0001).

**FIG 3:**
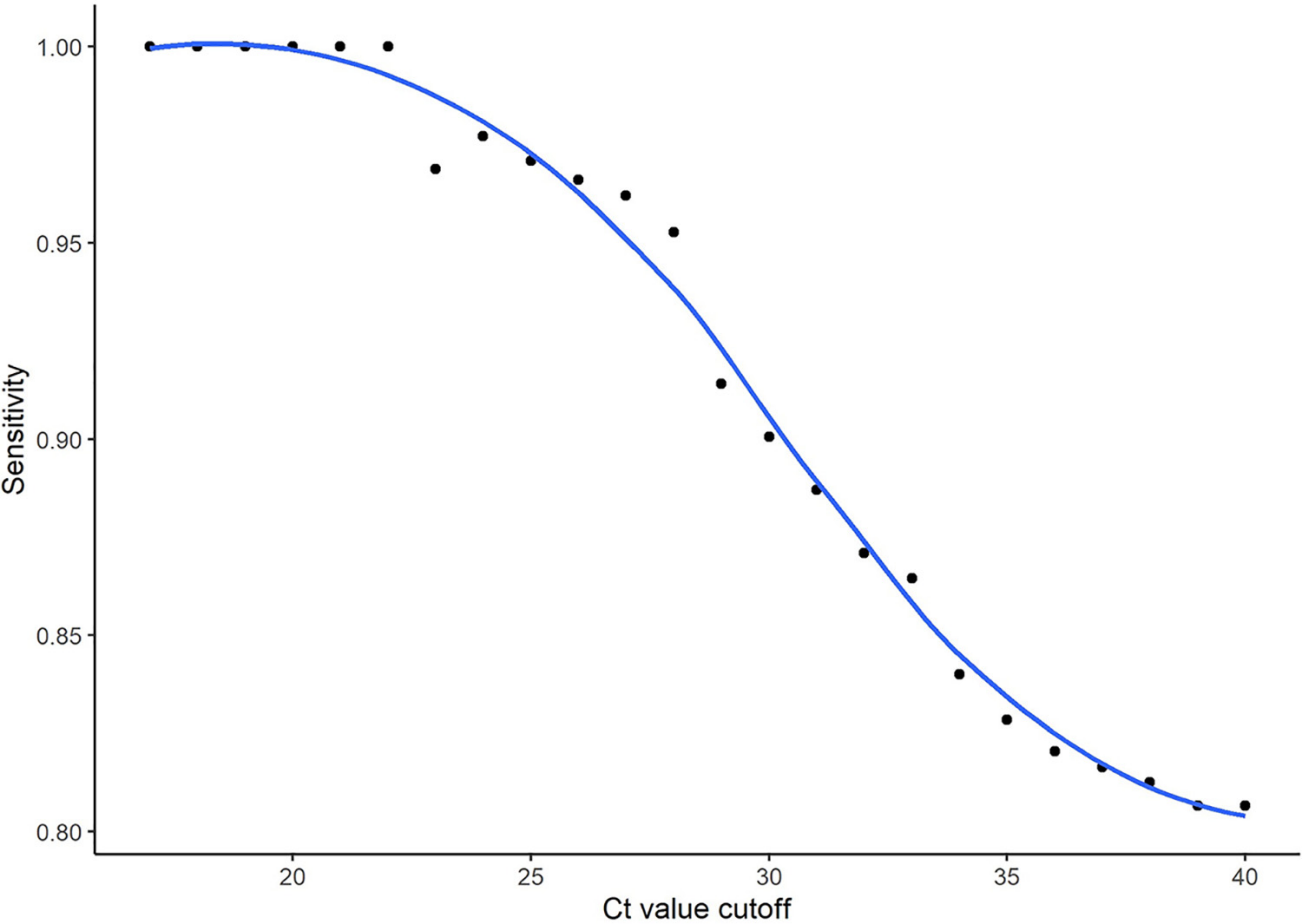
Graph showing rapid antigen sensitivity for different RT-PCR cycle threshold cutoff values. CT, cycle threshold.

The median cycle threshold (CT) count for all participants was 25 (24.5 for symptomatic and 27 for asymptomatic participants) (Fig. 2). Using this cutoff, the rapid antigen test sensitivity for participants with a CT count <25 was higher (97%) than for those with a CT count ≥25 (61%) (*P*  <  0.0001). This pattern was consistent between symptomatic and asymptomatic groups. Results show that sensitivity decreased as CT count increased (Fig. 2 and 3).

## DISCUSSION

This prospective study at a high-throughput, state-owned, self-referred walk-up COVID-19 testing site represents, to our knowledge, the largest evaluation of a rapid antigen test using BinaxNOW tests performed by health care workers. We demonstrated that mass testing sites can deploy rapid antigen test with minimal increase in staff. The BinaxNOW rapid antigen test had high accuracy in symptomatic individuals and adequate accuracy in some asymptomatic individuals. The sensitivity was 87% and, despite low COVID-19 prevalence, positive predictive value was 95% for symptomatic individuals, with the vast majority of symptomatic participants being tested within 7 days after symptom onset. The test sensitivity was 71% for asymptomatic individuals and the specificity was >99% for all individuals. The sensitivity was even higher for asymptomatic individuals who reported COVID-19 exposure and for participants who were considered more infectious based on their RT-PCR CT count. A total of 0.5% of the tests were indeterminate, which could be related to poor sampling or test kit–related issues. Our study demonstrates rapid antigen test sensitivity in symptomatic individuals above the 80% threshold set by the U.S. Food and Drug Administration for emergency use authorization (11). While rapid antigen test sensitivity is below this threshold in asymptomatic individuals, the test could still be considered for screening with serial testing. The U.S. Food and Drug Administration suggests, regarding emergency use authorization, “strategies for serial testing with less sensitive tests, such as 70% sensitivity, could increase overall sensitivity and be considered cumulatively rather than based on one-time testing” (11). Despite the high negative predictive value, patients with negative results represent a large proportion of total positive cases. Hence, a negative result does not preclude infection from a clinical and public health perspective.

Two large studies of BinaxNOW found lower sensitivity than we did, despite higher rates of asymptomatic COVID-19 prevalence (8, 12). However, Okoye et al. (12) reported results for samples that were self-collected by college students under supervision of trained nonmedical staff. Pollock et al. (8) reported accuracy results similar to ours. They also noted significantly lower test accuracy at temperatures below the manufacturer recommendations, and that all false positives were clustered in a single lot in the pediatric arm of the study. Their findings are consistent with ours in showing better sensitivity with lower CT counts. Hence, testing in the appropriate population (e.g., testing within 7 days after developing symptoms per the emergency use authorization) is important. A negative rapid test should be followed up with an RT-PCR test for symptomatic individuals with high clinical suspicion (7–9, 12–14).

Our study benefitted from a rigorous expert tester-driven training protocol for all testing providers, which could have contributed to the high level of accuracy. Sample collection technique, ambient conditions (such as temperature and humidity) of the storage and test site, variation in test kit quality between lots, and differences in patient characteristics may also explain differences in accuracy reported by the studies. Some of these factors, such as test administration by untrained individuals, may also play a role in test accuracy for the BinaxNOW COVID-19 Ag Card 2 Home Test (Abbott Laboratories), which was recently authorized for emergency use.

The accuracy of the rapid antigen test in the asymptomatic population suggests that repeat testing can be useful among asymptomatic individuals in congregate settings. Twice-per-week antigen testing has shown 95% sensitivity in detecting COVID-19 infection (15). Modeling by Paltiel et al. (16) of a hypothetical cohort of 5,000 college-age students suggested that frequent screening every 2 days using a test with a minimum 70% sensitivity and 98% specificity could contain outbreaks and support safe reopening. Our study found that the BinaxNOW rapid antigen test meets this 70% threshold and may offer a practical alternative to RT-PCR in this population when RT-PCR is unavailable.

Finally, our results indicate that rapid testing may facilitate timely referral for clinical evaluation, monitoring, or monoclonal antibody therapy. Such treatments (bamlanivimab/etesevimab; casirivimab/imdevimab) currently have emergency use authorization for individuals with mild disease and high risk of progression to severe disease and hospitalization. Monoclonal antibody treatments are authorized for administration only within a 10-day window after symptom onset and are likely of the greatest benefit during the first couple of days after symptom onset (17). A rapid antigen test could facilitate timely referral and treatment.

This study was performed at one high-volume center. Any potential systematic errors in testing administration or interpretation may have been missed by our quality control measures. Furthermore, our sample population was limited to adults and one urban location with a low SARS-CoV-2 prevalence of 3.7% among those tested. Importantly, we included individuals of diverse racial backgrounds. Additionally, because our testing staff has administered nearly 70,000 tests, our findings may not be generalizable to lower-volume centers with less skilled testing staff, such as schools or colleges. Not all tests were interpreted by two independent test readers. However, the concurrence rate in samples reviewed by a second tester was 100%, and our quality control mechanisms exceeded the manufacturer’s recommended process. An additional limitation is the narrow age range of the study participants (interquartile range, 26–41 years).

As one of a few large-scale studies assessing the performance of a rapid antigen test, our study provides guidance for public health practice and future research related to COVID testing. Our study further supports the utility of rapid antigen tests to meet volume targets needed to contain outbreaks and to safely reopen the economy (8, 9). Rapid antigen tests could be considered for screening asymptomatic people when a 24-h turnaround RT-PCR is unavailable, especially for repeat testing in congregate settings. When screening asymptomatic individuals, rapid antigen testing can help enable early identification and isolation, as well as enable timely referral for monoclonal antibody treatment in symptomatic individuals. Rapid antigen tests may also have a role in narrowing disparities in access to testing and RT-PCR testing wait times for patients of color if deployed in underserved communities. Further studies are needed to better understand variation in test accuracy across different conditions and home tests. Additional studies in pediatric populations will also be helpful in guiding testing in schools.

## MATERIALS AND METHODS

### Study design.

This was a single-center prospective study comparing the performance (measured using sensitivity and specificity) of the BinaxNOW rapid antigen test with the current gold standard of RT-PCR testing. BinaxNOW is a lateral flow assay that detects the SARS-CoV-2 viral nucleocapsid (N) protein. All samples for RT-PCR were sent to the University of Maryland Pathology Associates-Maryland Genomics reference laboratory (University of Maryland School of Medicine, Baltimore, MD) using a modification of the Centers for Disease Control and Prevention (CDC) 2019 Novel Coronavirus Real-Time RT-PCR Diagnostic Panel. The assay includes a panel of primer/probe sets targeting the viral N gene. This study was approved by the local institutional review board (Protocol # IRB00270236).

### Participants and study site.

Patients were tested at a high-volume walk-up community collection site at the Baltimore Convention Center Field Hospital during 10 consecutive testing days from December 23, 2020 to January 11, 2021. This testing site is part of the state-owned Baltimore Convention Center Field Hospital that is managed jointly by the University of Maryland Medical System and Johns Hopkins Medicine. In addition to being the largest COVID-19 testing site in the state, the field hospital is the longest running inpatient alternative care site in the country, the largest COVID-19 monoclonal antibody infusion treatment site in the state, as well as one of the mass COVID-19 vaccination sites in Maryland.

All adults arriving for walk-up RT-PCR testing were provided with written and verbal information about the study and given the opportunity to opt out of the additional anterior nares swabs for the rapid antigen test.

### Data collection.

At registration, before sample collection, information on symptom and exposure status was collected from each individual. For symptom status, individuals were asked questions according to the standard CDC symptom checklist (18). Those who reported at least one symptom on the checklist were considered “symptomatic,” and those who reported no symptoms on the checklist were considered “asymptomatic.” Additionally, patient exposure status was obtained according to CDC risk stratification. Patients were asked whether they lived with someone with confirmed or suspected COVID-19; if they had been within 6 feet of someone with confirmed or suspected COVID-19 for >15 min; and if they had had any other exposure to someone with confirmed or suspected COVID. The “no exposure” group reported no exposures to anyone with confirmed or suspected COVID-19. Date of symptom onset and exposure date was also obtained.

### Testing implementation, education, and training.

Tests were administered in a 32,000-ft^2^ indoor hall, re-engineered for improved airflow and filtration. The hall was kept at 72°F and <50% humidity and had space for up to 200 physically distanced individuals. Two lanes with six testing stations each (total of 12 stations) were set up with a tester and a testing assistant at each station. The tester obtained the sample, and the testing assistant helped label and process it. Twenty-four testing staff were needed for testing. The site was open for 6 h each day on 6 days per week (closed on Sundays). For this study, an additional “reading station” was set up for each lane and staffed by one test reader. The testing assistant time-stamped each rapid antigen kit and moved it to the reading station where the results were read and recorded within the time frame specified by the manufacturer.

Testers and assistants were qualified to test or assist after passing testing competencies for RT-PCR testing and completing a credentialing checklist. Once testers and assistants successfully completed competencies, they were given additional training for the rapid antigen assay if they were not previously trained in administering the BinaxNOW rapid antigen test. Expert testers with experience in using the BinaxNOW rapid antigen test were responsible for training additional testing staff. Expert testers received virtual training from an Abbott BinaxNOW technical manager or from an expert tester who had received this training previously and administered multiple tests at another site. Training occurred daily at the beginning of testing sessions.

BinaxNOW rapid antigen training for testing staff comprised the following steps:

1. Trainee staff members reviewed the manufacturer’s instructions for the BinaxNOW COVID-19 antigen card, including examples of positive, negative, and indeterminate results.
2. Trainees observed expert testers administering the test.
3. Trainees administered the test while being observed by the expert testers.
4. After expert testers determined that the trainee adequately administered the test, trainee staff members were considered qualified to administer tests independently.

### Quality control.

Trained testing staff providers performed test kit quality control checks on each box of 40 procedure cards before use, per the manufacturer’s instructions. On 6 of 10 study testing days, random test read quality control checks were performed by expert testers while staff read the rapid antigen test results (negative, positive, or indeterminate). The expert testers reviewed the results of the rapid antigen test and noted whether they concurred with the testing staff.

### Sample collection.

The rapid antigen and RT-PCR samples were collected sequentially for each participant in the study by medical staff who were trained to administer rapid antigen tests, as described earlier. Per the manufacturer’s guidelines, rapid antigen samples were collected from the bilateral anterior nares and immediately inserted into the test card. A designated, trained reader interpreted and recorded the result on-site for each rapid antigen test 15 min after the test was administered per the instructions for use. Individuals for whom the RT-PCR or rapid antigen test was deemed indeterminate (control line not interpretable) were excluded from analysis.

### Statistical analysis.

Accuracy results (sensitivity, specificity, and positive and negative predictive values) and 95% confidence intervals were calculated using the binomial exact method for the rapid antigen test for both symptomatic and asymptomatic populations compared with the RT-PCR gold standard. Accuracy results for symptomatic and asymptomatic group were compared. Two-tailed *P values* were calculated using Fisher’s exact test. Test accuracy was calculated for each exposure group. A beeswarm plot showing the distribution of RT-PCR CT values for true positive and false positive rapid antigen test results was generated for symptomatic and asymptomatic individuals. All statistical analyses were performed using JMP Pro, version 14.0.0, software (SAS Institute, Cary, NC).
