# Supplementary material for: Implementation and Accuracy of BinaxNOW Rapid Antigen COVID-19 Test in Asymptomatic and Symptomatic Populations in a High-Volume Self-Referred Testing Site
Source: Microbiol Spectr. 2021 Dec 1;9(3):e01008-21. doi: 10.1128/Spectrum.01008-21 (PMC8668078; doi:10.1128/Spectrum.01008-21)
Supplement: SUPPLEMENTAL FILE 1 — Supplemental material. Download SPECTRUM01008-21_Supp_1_seq7.pdf, PDF file, 0.1 MB [file spectrum01008-21_supp_1_seq7.pdf]

Appendix 1: Checklist Used to Determine COVID Testing Competency for Testing Staff

COVID Testing Competency

**TESTER**	**INITIALS**
Greet client	
Verify: Name	
Date of Birth	
Address	
Test Ordered	
If corrections are needed notify Navigator for assistance	
Explain process to client	
Offer client hand sanitizer, do not have them to touch any item on the table to minimize contamination	
Have client lower facemask and blow nose to loosen mucus	
Have client place facemask over mouth below nose	
Position client: Sitting in chair	
Head in neutral position looking straight ahead	
Perform test	
Nasopharyngeal	
i. Insert swab parallel to floor until resistance met – approximately 2-2.5 inches (close to the break point of the swab)	
ii. Wait 10 seconds	
iii. Carefully rotate swab 3 times	
iv. Remove swab and place in Specimen Tube	
Nasal Swab	
i. Insert at least 1-1.5 inches into nares	
ii. Swab interior of each nares at least 5 times	
iii. Remove swab and place in Specimen Tube	
Offer client tissue and hand sanitizer	
Dispose outer gloves	
Use hand sanitizer to clean gloved hands	
Use wipe to clean table, items touched, and chair	
Remove outer gloves and dispose of	
Use hand sanitizer on gloved hands	
Don outer gloves to begin again	

Educator/Observer: ______________________ **Date:** ________

Employee Signature: ____________________________________
